# Long-term mTOR inhibitors administration evokes altered calcium homeostasis and platelet dysfunction in kidney transplant patients

**DOI:** 10.1111/jcmm.12044

**Published:** 2013-04-12

**Authors:** Esther López, Alejandro Berna-Erro, Nuria Bermejo, José María Brull, Rocío Martinez, Guadalupe Garcia Pino, Raul Alvarado, Ginés María Salido, Juan Antonio Rosado, Juan José Cubero, Pedro Cosme Redondo

**Affiliations:** a Cell Physiology Research Group, Department of Physiology, University of ExtremaduraCáceres, Spain; b Department of Renal Transplantation, Infanta Cristina HospitalBadajoz, Spain; c Department of Hematology, San Pedro de Alcantara HospitalCáceres, Spain; d Hematology division, Extremadura County Blood Donation CenterMérida, Spain

**Keywords:** Platelets, rapamycin, calcium, mTOR, thrombosis

## Abstract

The use of the mammal target of rapamycin (mTOR) inhibitors has been consolidated as the therapy of election for preventing graft rejection in kidney transplant patients, despite their immunosuppressive activity is less strong than anti-calcineurin agents like tacrolimus and cyclosporine A. Furthermore, as mTOR is widely expressed, rapamycin (a macrolide antibiotic produced by *Streptomyces hygroscopicus*) is recommended in patients presenting neoplasia due to its antiproliferative actions. Hence, we have investigated whether rapamycin presents side effects in the physiology of other cell types different from leucocytes, such as platelets. Blood samples were drawn from healthy volunteers and kidney transplant patients long-term medicated with rapamycin: sirolimus and everolimus. Platelets were either loaded with fura-2 or directly stimulated, and immunoassayed or fixed with Laemmli's buffer to perform the subsequent analysis of platelet physiology. Our results indicate that rapamycin evokes a biphasic time-dependent alteration in calcium homeostasis and function in platelets from kidney transplant patients under rapamycin regime, as demonstrated by the reduction in granule secretion observed and subsequent impairment of platelet aggregation in these patients compared with healthy volunteers. Platelet count was also reduced in these patients, thus 41% of patients presented thrombocytopenia. All together our results show that long-term administration of rapamycin to kidney transplant patients evokes alteration in platelet function.

## Introduction

Mammalian target of rapamycin (mTOR) is a serine/threonine kinase downstream of Akt/PKB that is activated either by intracellular second messengers or receptor-associated kinases like insulin receptors 1–4. Two mTOR complexes have been identified and, they are designated as mTOR complex 1 (mTOR1) or mTOR complex 2 (mTOR2) 1, 5, 6, involving the proteins raptor and rictor respectively. mTOR1/2 resulting complexes regulate different downstream pathways by phosphorylation. For instance, mTOR1 impairs protein phosphatase 2A activity 7 and, contrary, it activates by phosphorylation the transcription factor activators 4EBP, HIF1α 8 and S6K 9. In addition, mTOR2, among other functions, regulates actin cytoskeleton reorganization by up-regulating PKC, Rho and Rac activities. Furthermore, mTOR2 has been described upstream of Akt/PKB. Hence, several key intracellular pathways require mTOR activity, being mTOR particularly relevant in the cellular cycle through the control of cell growing, proliferation and apoptosis; therefore, it often represents a good target to prevent neoplasia and other illnesses 10.

Some investigations have revealed that rapamycin is neither so good nor specific mTOR inhibitor, as its administration would inhibit mTOR1 upon complexing with several members of the immunophilin family, like FKBP12 or FKBP52 11–13. By contrast, mTOR2 complex activity would remain unaltered in the presence of the drug, unless that high concentrations or chronic administration are used 1, 14. Furthermore, rapamycin complexing to immunophilins might be involved in the activation of calcium-ATPases, like the sarcoendoplasmic Ca^2+^-ATPase (SERCA) 15, 16, and plasma-membrane Ca^2+^-ATPase 4C, as well as the inositol 1,4,5-trisphosphate receptor type I in neurons 17, 18.

Several new potent drugs have been designed nowadays and some mTOR inhibitors showed satisfactory immunosuppressor activity, like everolimus 19. Nevertheless, sirolimus (rapamycin) is the therapy of election to prevent graph rejection in kidney transplant patients, where renal function has been compromised owing to previously administration of other immunosuppressors that target calcineurin, such as CsA or tacrolimus 20, 21.

Hence, we have explored here the possible side effects of two mTOR inhibitors, sirolimus and everolimus, in platelets from kidney transplant patients long-term medicated with mTOR inhibitors.

## Materials and methods

### Materials

Fura-2 acetoxymethyl ester (Fura-2/AM) was from Molecular Probes (Leiden, The Netherlands). Apyrase (grade VII), aspirin, bovine serum albumin (BSA), dithiothreitol (DTT), quinacrine, adenosine 5′-diphosphate (ADP) and thrombin (Thr) were from Sigma-Aldrich (Madrid, Spain). Tert-Butyl hydroquinone (TBHQ) was from Alexis (Nottingham, UK). Anti-CD62P-PE antibody, anti-CD41-a PerCP (clone HIP8) and anti-PE isotype were from Becton Dickinson Transduction Laboratories (Madrid, Spain). Anti-phospho-mTOR (Ser 2481) and anti-phospho-raptor (Ser 722) antibodies were from Millipore (Hayward, CA, USA). Anti-phospho-Akt (Thr 308) antibody was from Cell Signalling technology (Beverly, MA, USA). Horseradish peroxidase-conjugated antimouse IgG antibody was from Amersham (Buckinghamshire, UK). Enhanced chemiluminescence detection reagents were from Pierce (Cheshire, UK). All other reagents were of analytical grade.

### Selection of patients, blood processing and platelet samples preparation

Kidney transplant patients and healthy volunteers were selected by the Department of Renal Transplantation of Infanta Cristina Hospital (Badajoz, Spain). Twenty nine kidney transplant patients ranging from 35 to 72 years old under sirolimus treatment (Rapamune® administered at 1.88 ± 0.5 mg/12 hr, *n* = 21 patients) or everolimus (Certican® administered at 1.81 ± 0.3 mg/24 hr, *n* = 8 patients), and administration of mTOR inhibitor was combined with daily administration of prednisone (up to 10 mg) and healthy volunteers of similar age range were selected (*n* = 6). A similar number of men (17 patients and 3 healthy volunteers) and women (12 patients and 3 healthy volunteers) have been considered in both patients and control groups included in the present investigation. Vascular or thrombotic problems were not diagnostized either before or after transplantation proceeds. Selected patients presented at the time of the study creatinin concentration and clearance rate of 1.64 ± 0.63 (mg/dl) and 61.93 ± 25.68 (ml/min.) respectively. The blood glucose values observed in the selected patients were 95.52 ± 15.82 (mg/dl). Two patients were excluded from the results during the study mainly as they required hospitalization and further surgical intervention, hence rapamycin treatment had to be removed previous to rehospitalization. Finally, at the time of blood extraction, trough level monitored of sirolimus and everolimus was 8.59 ± 2.34 and 6.75 ± 1.27 ng/ml respectively.

Upon informative consents were given according to Helsinki's declaration, early morning blood samples were drawn by venipuncture during common patients controls (performed by qualified staff) using vacutainer tubes with 6.3 mg EDTA-K3 to prevent coagulation. The tubes and sampling procedure have been demonstrated to keep platelet size and other platelet parameters within the 180 min. after blood drawn 22. One of the tubes extracted was used for evaluating general wellness parameters, like trough levels of sirolimus and everolimus, creatinine clearance rate, plasma creatinine concentration, platelets count and volume and blood glucose concentration. The second tube was supplemented with apyrase alone (40 μg/ml) or in combination with aspirin (100 μM), and used for platelet calcium homeostasis and granule secretion determinations. All determinations were done during the following 3–4 hr from blood extraction.

### Measurement of cytosolic-free calcium concentration ([Ca^2+^]_c_)

Fura-2-loaded platelets were prepared as described previously 23–25. Platelet-rich plasma obtained upon sequential centrifugation was incubated at 37°C with 2 μM fura-2/AM for 45 min. Cells were then collected by centrifugation at 350 × *g* for 20 min. and resuspended in HEPES-buffered saline (HBS) containing (in mM): 145 NaCl, 10 HEPES, 10 D-glucose, 5 KCl, 1 MgSO_4_, pH 7.40 and supplemented with 0.01% w/v bovine serum albumin and 40 μg/ml apyrase.

Fluorescence was recorded from 1.0 ml of platelet suspension aliquots (2 × 10^8^ cells/ml) using a fluorimeter (Cary Eclipse, Varian, Madrid, Spain). Monitored fluorescence records were transformed into cytosolic-free calcium concentrations ([Ca^2+^]_c_) using the fura-2 340/380 fluorescence ratio and calibrated according to the method of Grynkiewicz 26.

### Determination of platelet granule content and secretion

Platelets were first gated by size (FSC) and complexity (SSC) and 8000 events were counted. α- and δ-granule secretion was monitored in CD41-gated platelets by monitoring fluorescence change in platelet samples using a flow cytometer (FASCcan cytometer; Becton-Dickinson, San Jose, CA, USA). Samples of 50 μl of plasma rich platelets (PRP) were suspended in 450 μl of tempered HBS and platelet δ-granules were stained by incubating at 37°C for 30 min. with 10 μM of the quinacrine fluorescence probe. The attenuation in quinacrine fluorescence of platelets is indicative of δ-granule secretion and it is expressed as mean fluorescence intensity (MFI = quinacrine fluorescence − endogenous fluorescence) 27–29. Meanwhile, α-granules secretion was monitored using a specific anti-P-selectin antibody (anti-CD62P-PE) 30. Incubation with anti-CD62P antibody was done for 10 min. upon cell stimulation with the physiological agonist thrombin (Thr), and incubation time was finished by mixing with ice-cold phosphate buffer saline. Fluorescence emitted by anti-CD62P-PE antibody and quinacrine was gated in cell positively stained with anti-CD41-a PerCP (clone HIP8) antibody that is indicative of positive platelet identification.

### Aggregometry

The percentage and delay time of aggregation was monitored from aliquots of 400 μl of washed platelets isolated from kidney transplant patients treated with either sirolimus and everolimus, using a Chronolog aggregometer (Havertown®, Havertown, PA, USA) at 37°C under stirring at 1200 r.p.m. 31. Percentage of aggregation was estimated as the percentage of the difference in light transmission between the platelet suspended in HBS and HBS alone, and it is shown as the percentage of platelet aggregated in response to Thr (0.1 U/ml) or ADP (10 μM), compared to resting platelets. HBS-free platelet medium is considered to be 100% of aggregation and resting platelets is arbitrarily 0%. The delay time is considered as the time required for reaching the maximum aggregation percentage in each platelet suspension.

### Western blotting

Western blotting was performed as described previously 32, 33. Briefly, 250 μl aliquots of platelet suspension (1 × 10^8^ cell/ml) were stimulated with Thr (0.1 U/ml) for 1 min. and fixed by mixing with equal volume of Laemmli's buffer (2×) using reducing conditions (5% final concentration of dithiotheitrol, DTT). Proteins were isolated in a 6% acrilamyde-bisacrilamide SDS-PAGE and separated proteins were electrophoretically transferred onto nitrocellulose membranes for subsequent analysis by Western blotting (WB). Blots were incubated overnight with blocking buffer, containing 5% (w/v) skimmed milk, to block residual protein-binding sites. Immunodetection of mTOR and evaluation of the phosphorylation state of mTOR and ractor activation were achieved using an anti-phospho-mTOR (Ser 2481, autophosphorylation residue) and phospho-raptor (Ser 722) antibodies 34, 35, overnight at 4°C and diluted 1:1000 in blocking buffer. The primary antibody was removed and blots were washed with Tris-buffered saline supplemented with tween 20 (TBST) six times for 5 min. To detect the primary antibodies, blots were incubated for 1 hr with the appropriate horseradish peroxidase-conjugated secondary antibody diluted 1:7500 in TBST [containing 5% (w/v)]. Membranes were then incubated with enhanced chemiluminescence reagent for 4 min. and they were subsequently exposed to photographic films. The density of bands on the film was measured using the Image J free software from national health institute of USA (NIH). Reprobing of the membranes with anti-actin antibody was done to assess that a similar amount of proteins was loaded in all gel lanes.

### Statistical analysis

Patients were included in four groups according to the time of administration of either sirolimus (six patients within each group) or everolimus (four patients within each group); hence, patients medicated less than 24 months were considered as group I. Group II were medicated during 24–36 months, Group III were medicated during 36–60 months and group IV were medicated over 60 months. Analysis of statistical significance was performed using Student's unpaired *t*-test. In addition, one-way anova was performed, and to evaluate differences between groups we used the Dunnett's test. Only values with *P* < 0.05 were accepted as significant.

## Results

### Altered calcium homeostasis in platelets from kidney transplant patients treated with sirolimus and everolimus

**Fig. 1:**
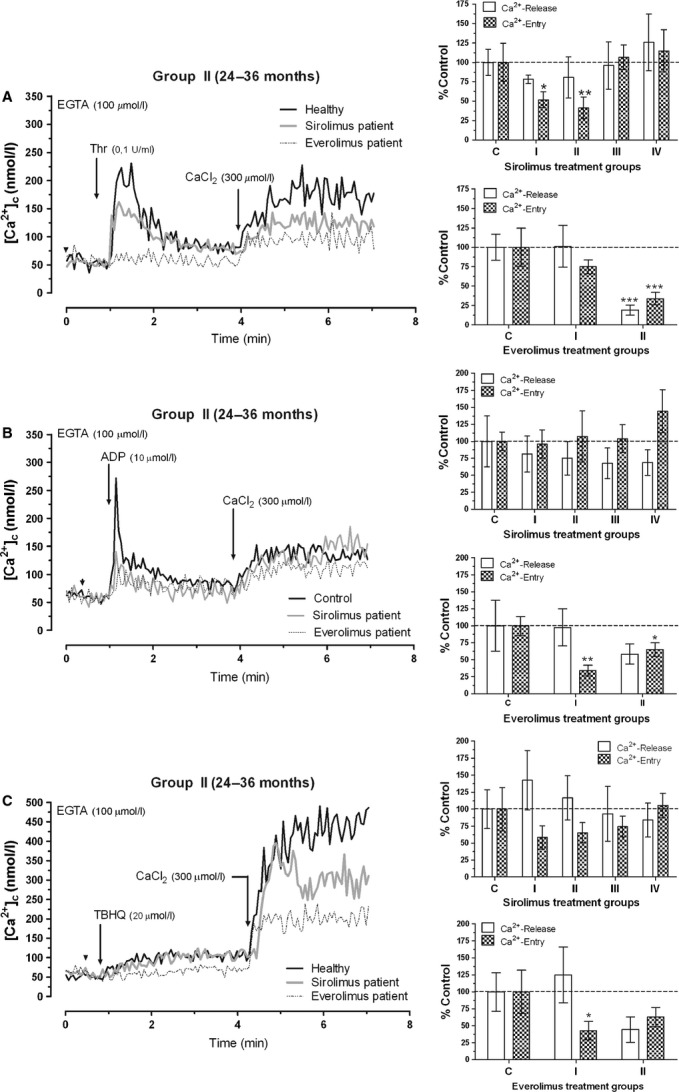
Calcium homeostasis in patients under sirolimus and everolimus medication. Fura-2-loaded platelets isolated from healthy (black solid lines) and patients treated with sirolimus (grey lines) or everolimus (black-doted lines), were suspended in Ca^2+^-free HBS medium (100 μM EGTA was added; arrowheads) and subsequently stimulated either with Thr (**A**), ADP (**B**) and THBQ (**C**) for 3 min., followed by addition of 300 μM of CaCl_2_ to the extracellular medium to initiate calcium entry. Representative calcium signals of patients belonging to group II are plotted and histograms on the right hand side, represent calcium release and entry as percentage of control of patients treated with sirolimus (*n* = 6 each group) and everolimus (*n* = 4 each group) and medicated during less than 24 (I), 24–36 (II), 36–60 (III) and over 60 months (IV). *, ** and ***, represents *P* < 0.05, <0.01 and <0.01 compared with healthy individuals respectively.

Correlation analysis performed in kidney transplant patients, revealed that sirolimus administration for long periods might alter calcium entry, being particularly affected the group II of patients (medicated for 24–36 months; Table 1), although trough levels of sirolimus are unlikely the key factors. As shown in Figure 1, fura-2-loaded platelets from patients and healthy individuals were suspended in a Ca^2+^-free HBS medium (100 μM EGTA was added), and were stimulated for 3 min. with thrombin (Thr; 0.1 U/ml; Fig. 1A) or ADP (10 μM; Fig. 1B) and then 300 μM CaCl_2_ was added to the extracellular medium to initiate calcium entry. Our results indicate that both Ca^2+^ release and entry in response to Thr were altered in most of the groups analysed, being most evident in group II of patients compared with healthy individuals (see Fig. 1A and 1B, where sirolimus reduced in Ca^2+^ entry evoked by Thr in a 59.8 ± 14.1% (*P* < 0.01; *n* = 6)). The effect of sirolimus on ADP-evoked Ca^2+^ mobilization was not so evident as presented for Thr, but it resulted in a small and time-dependent increase in Ca^2+^ release among the different patient groups as compared with healthy individuals. Meanwhile, reduced Ca^2+^ entry in these groups was observed, and despite this difference was not statistically significant a clear tendency was found [32.9 ± 28.0% (*P* > 0.05; *n* = 4) in group II]. The different effect on Thr- and ADP-evoked Ca^2+^ signals might be explained because of the fact that ADP releases Ca^2+^ from the dense tubular system (DTS; similar to the endoplasmic reticulum in other cells) and Thr mobilizes calcium from the DTS and the acidic stores 36.

**Table 1:** Correlation analysis of demographic and physiological variables of kidney transplanted patients administered sirolimus and everolimus. Grey background boxes highlights variables that are significantly correlated

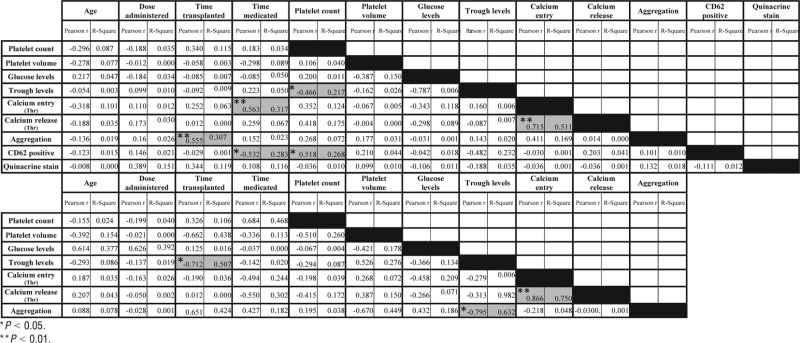

On the other hand, tert-butyl hydroquinone (TBHQ) releases Ca^2+^ from the acidic stores in platelets 36–38. As shown in Figure 1C, in group II of patients, sirolimus induced a reduction of 34.9 ± 14.9% in TBHQ-evoked Ca^2+^ entry (*P* > 0.05; *n* = 6). Hence, considering that ADP-evoked Ca^2+^ entry resulted unaltered, we suggest that sirolimus mostly affects SOCE controlled by acidic granules.

Treatment with everolimus altered Ca^2+^ homeostasis evoked by Thr, ADP and TBHQ (Fig. 1, see graphs and right hand side histograms). We have found that in the group II of patients (which received everolimus for more than 24 months), Ca^2+^ release was reduced by 80.8 ± 6.3% (*P* < 0.001; *n* = 4), 41.7 ± 14.7% (*P* > 0.05; *n* = 4) and 55.6 ± 18.97% (*P* > 0.05; *n* = 4) in platelets stimulated with Thr, ADP and TBHQ respectively. Similarly, Ca^2+^ entry was reduced by 66.1 ± 8.0% (*P* < 0.001; *n* = 4), 35.2 ± 10.1% (*P* < 0.05; *n* = 4) and 37.0 ± 14.0% (*P* < 0.05; *n* = 4) in platelets stimulated with Thr, ADP and TBHQ respectively.

### Sirolimus evokes reduction in platelet granule secretion from kidney transplant patients

**Fig. 2:**
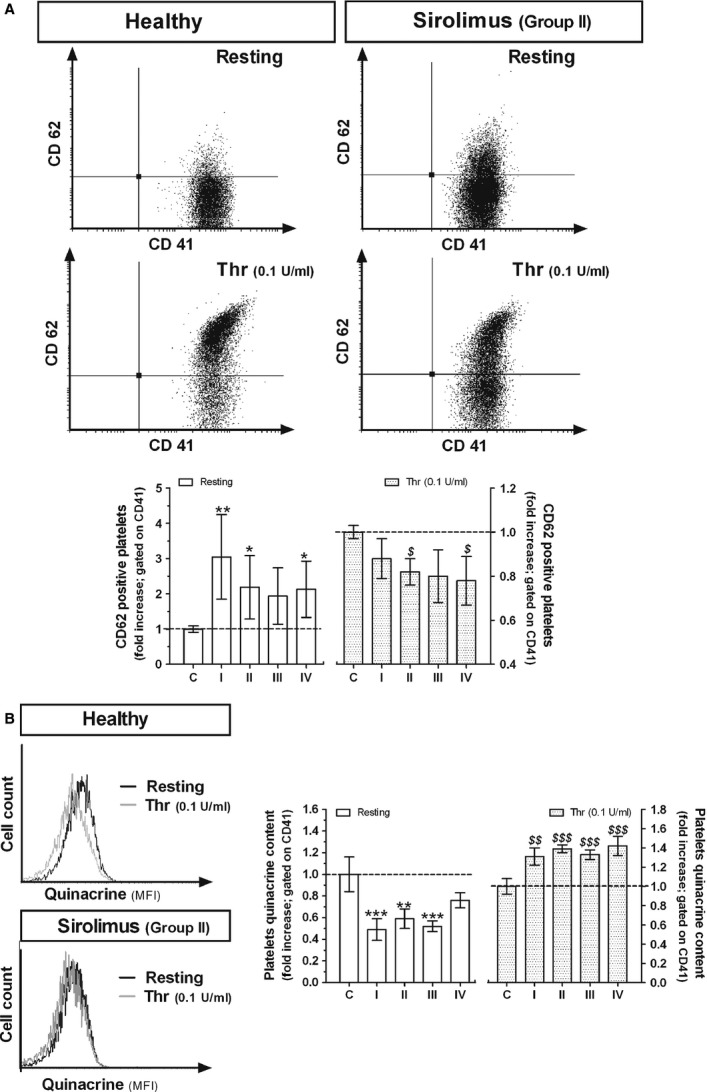
Granule secretion in patients medicated with sirolimus. Platelets isolated from kidney-transplanted patients treated with sirolimus were incubated in plasma rich platelets (PRP) for 30 min. at 37 °C with anti-CD41 and quinacrine (10 μM). Platelets were then left under resting condition or stimulated for 10 min. with Thr (0.1 U/ml), and simultaneously, anti-CD62 antibody (P-selectin; diluted 1:50) was added to medium. Fluorescence of CD41 was used for selecting platelet positive cells. Fluorescence was analysed using flow cytometry, from patients under sirolimus medication for different periods of time (<24: I), (24–36: II),(36–60: III) and (>60 months: IV). The fluorescence results of recording CD41 and CD62-positive platelets (**A**; α-granules) and simultaneously, CD41 and quinacrine (**B**; δ-granules) in the same platelets sample. Histograms show either the increase in P-selectin membrane exposition or quinacrine fluorescence remaining in the platelets in fold increase. *, ** and ***, represent *P* < 0.05, <0.01 and <0.01 compared with values found in resting platelets from healthy individuals (*n* = 6). While, ^*$*^, ^*$$*^, ^*$$$*^, represent *P* < 0.05, <0.01 and <0.01 compared with Thr-stimulated values found in healthy individuals.

Ca^2+^ homeostasis regulates several intracellular mechanisms in human platelets like actin cytoskeleton reorganization, shape change or granule secretion. Hence, using flow cytometry, we gated CD41+ cells (platelet positive staining), and fluorescence of anti-P-selectin (CD62P) antibody and quinacrine was monitored. Fluorescence protocols have been widely used to evaluate alpha (α-) and dense (δ-) granule secretion 39. As shown in Figure 2A, platelets present low levels of surface-exposed P-selectin under resting conditions (Fig. 2A; C: white bars representing resting platelets from healthy individuals), which is drastically enhanced upon α-granule secretion stimulated by Thr. Furthermore, we found that sirolimus-treated patients presented enhanced P-selecting membrane exposure under resting conditions, and subsequently, Thr-evoked P-selectin exposure was significantly lower (*P* < 0.001; *n* = 6); thus, the reduction in the fold increase observed between platelets from the group II of patients treated with sirolimus compared with control was of 0.18 ± 0.06 (Fig. 2A, right-hand side histogram; *P* < 0.05; *n* = 6). P-selecting exposition reached a 4.6 ± 0.1 fold increase (*P* < 0.001; *n* = 6) in Thr-stimulated platelets from healthy individuals. Hence, α-granule secretion was altered by sirolimus in a time-dependent manner (Fig. 2A, right-hand side histogram). Regarding everolimus patients, the most samples in resting conditions presented a very high elevated P-selectin exposure under resting condition, which makes subsequent evaluation of granule secretion difficult.

In addition, in healthy individuals, Thr (0.1 U/ml) reduced quinacrine staining by 1.6 ± 0.04 fold decrease respect to the fluorescence found in platelets under resting conditions (*P* < 0.05; *n* = 4). Thr-evoked δ-granules secretion, and subsequently, lost of quinacrine fluorescence. Upon platelets stimulation with Thr quinacrine stain remaining inside the platelets was higher in patients treated with sirolimus than in control, owing to the inhibition of granule secretion. Thus, a 1.3 ± 0.10 fold increase (*P* < 0.01; *n* = 4), 1.4 ± 0.04 (*P* < 0.001; *n* = 4), 1.3 ± 0.05 (*P* < 0.001; *n* = 4), 1.4 ± 0.10 (*P* < 0.001; *n* = 4) was observed in groups I, II, III and IV patients treated with sirolimus respectively (Fig. 2B). As it has been shown for α-granule secretion, δ-granule secretion resulted higher in platelets from patients under resting condition compared with platelets from healthy individuals.

### Long-term administration of sirolimus and everolimus significantly alter platelet aggregation in response to physiological agonists

**Fig. 3:**
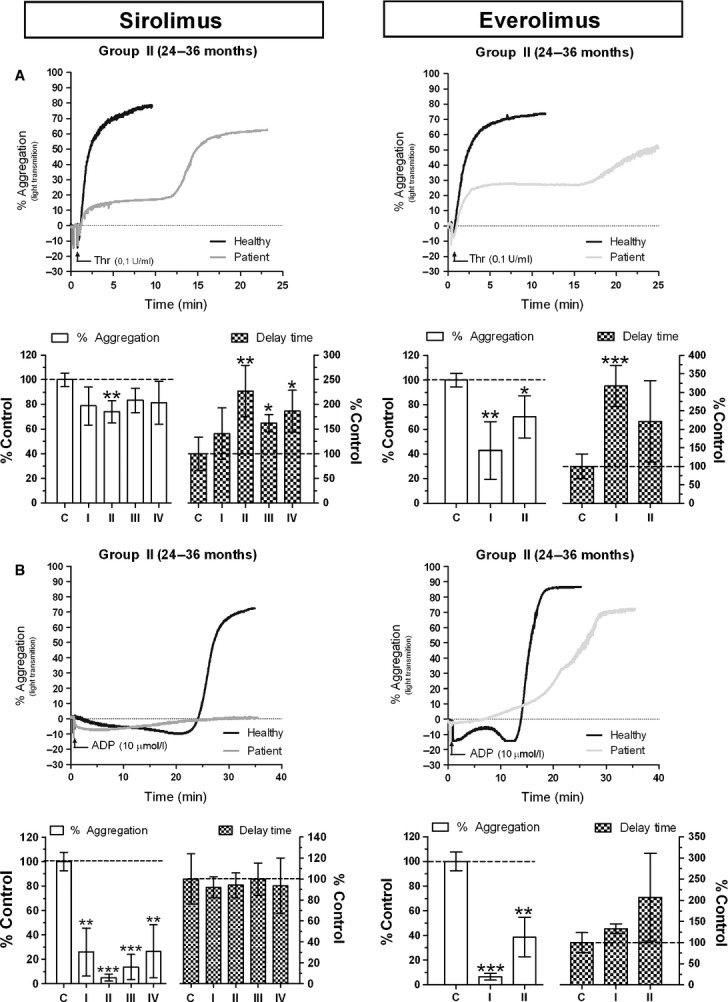
Sirolimus and everolimus alter aggregation in long-term medicated patients. Aliquots of platelets (400 μl) were suspended in fresh rich Ca^2+^-free HBS medium (1 mM), and then, they were stimulated with 0.1 U/ml of Thr (**A**) or 10 μM of ADP (**B**) to determine platelet aggregation as it is described in Materials and Methods. Histograms represent the percentage of aggregation and delay time, considering the latest as the time required to reach maximum percentage of aggregation, compared with healthy individuals. *, ** and ***, represent *P* < 0.05, <0.01 and <0.01 compared with healthy individuals, respectively.

Aggregation is a process finely regulated by, among others, Ca^2+^ homeostasis, protein phosphorylation, and other events like surface exposure of molecules, such as P-selectin (CD62P) or tetraspanin (CD63), which favour platelet–platelet and platelet–endothelium interaction 40. As shown in Figure 3, sirolimus and everolimus administration perturbed platelet aggregation in response to Thr and ADP, as demonstrated by observing the percentage of aggregation and, even more evidently, by evaluating the delay time, which is considered as the time required to reach the maximum percentage of aggregation in each platelet sample. Percentage of aggregation was significantly reduced in group II of patients treated with sirolimus (by 26.1 ± 8.8% compared with healthy individuals; Fig. 3A; *P* < 0.01; *n* = 6). The decrease in percentage of aggregation was accompanied of an increase of 227.2 ± 52.0% (*P* < 0.01; *n* = 6) in the delay time compared with healthy individuals where it never exceeded 6.8 ± 2.3 min. (*P* < 0.01; *n* = 6). In the case of everolimus, Thr-evoked aggregation was also found reduced by 57.2 ± 2.3% (*P* < 0.01; *n* = 6) in patients treated for less than 24 months (group I), while the delay time was enhanced by 317.6 ± 55.6% (*P* < 0.001; *n* = 6) as compared with platelets from healthy individuals. Furthermore, sirolimus caused greater alterations in ADP-evoked aggregation in the group II of patients (Fig. 3B, 94.8 ± 3.0%; *P* < 0.001; *n* = 6); meanwhile everolimus mostly affected the group I patients (93.4 ± 2.7%; *P* < 0.001; *n* = 6).

### Sirolimus reduces phosphorylation by altering mTOR activation in human platelets

**Fig. 4:**
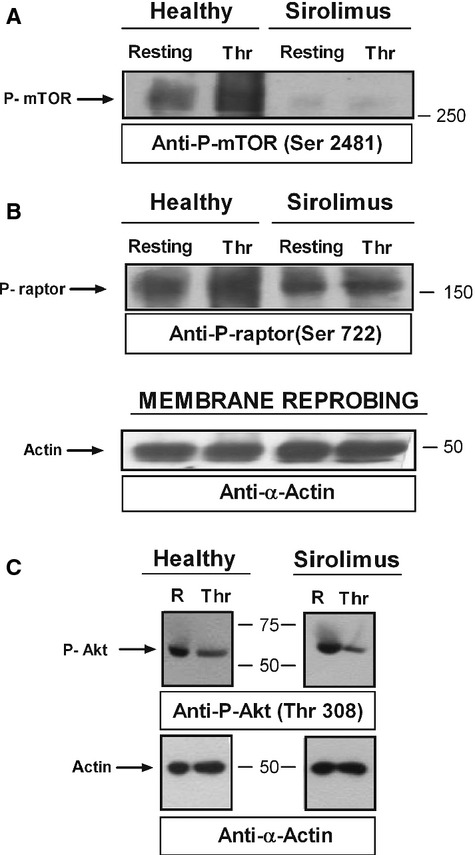
Long-term administration of sirolimus evokes reduction in phosphorylation of proteins belonging to mTOR signalling cascade. Platelets from healthy individual or sirolimus-treated patients (during 24–36 months) were stimulated in Ca^2+^-free HBS medium for 1 min. with Thr and then fixed in 2xLB (5% final DTT concentration). Western blotting was performed using anti-phospho-mTOR (Ser 2481; **A**), anti-phospho-raptor (Ser 722; **B**), anti-phospho-Akt (Thr 308; **C**) antibodies, all of them diluted (1:1000) in blocking buffer containing skimmed milk. Specific secondary antibody was used to develop the membranes as described under Material and methods. Membranes were reprobed using an anti-α-actin antibody to corroborate that similar amount of proteins were loaded in each lane. Panels are representative of four independent experiments using samples belonging to group II of patients and healthy individuals.

As shown in Figure 4, Thr stimulation evokes an increase in mTOR phosphorylation in platelets from healthy individuals, and subsequently, as a result of an enhanced mTOR activation an increased phosphoserine levels of raptor was observed. As expected, and it is shown in Figure 4A and B (representative experiment of patients belonging to group II of patients is shown) the phosphorylation levels of both mTOR and raptor were attenuated in patients that were long-term treated with sirolimus.

To ascertain whether these changes in proteins belonging to mTOR complex might affect to the activity of the mTOR complex, we have investigated the phosphorylation state of Akt, which has been described belonging to the same mTOR signalling pathway, and it has been reported to be crucial during platelet activation. As presented in Figure 4C, Akt resulted dephosphorylated during the initial steps of the platelet activation with Thr (0.1 U/ml), which agrees with previous observation in other cells type upon G protein-coupled receptor activation, like thrombin receptor or colecystokinin receptor 41. Akt phosphorylation pattern was not significantly altered in presence of rapamycin upon stimulation with Thr as previously reported in platelets 42. Membranes were reprobed using anti-α-actin antibody to asses that similar amount of proteins have been loaded in all lanes.

### Correlation analysis of different variables in kidney transplant patients receiving long-term administration of sirolimus and everolimus

To further explore the possible impairment of platelet function by administration of mTOR inhibitors, several variables were analysed in the different patient groups. As shown in Table 1, we found positive correlation between the *time transplanted* and *platelet aggregation* in patients treated with sirolimus (*P* < 0.05; *n* = 19), indicating that platelets aggregation from transplanted patients recovered functionality 5 years after the transplant. Interestingly, aggregation values were found similar to those in healthy individuals. We have also observed correlation between the *time medicated* and *Ca*^*2+*^ *entry* (*P* < 0.01; *n* = 21). Interestingly, patients with smaller *platelet count* presented also reduced platelet α-*granule secretion* in response to Thr (*P* < 0.05; *n* = 16), which might be indicative of a greater clearance rate of pre-stimulated platelets in these patients. Furthermore, we observed negative correlation between *time medicated* and platelet α-*granule secretion* (*P* < 0.05; *n* = 16), and between *trough levels* of sirolimus and *platelet count* (R squared: 0.2196; *P* < 0.05; *n* = 21). Regarding the rest of correlations analysed, none of them presented R squared values high enough to result statistically significant (see Table 1). Furthermore, we have observed that patients presenting higher trough levels of mTOR inhibitors, also showed significant reduction in platelet count, which might be indicative of a lower platelet generation from bone marrow or higher rate of platelet clearance.

On the other hand, patients treated with everolimus presented lower rate of correlation among the variables considered in this study. We found negative correlation between time transplanted and trough levels (*P* < 0.05; *n* = 8), and also between trough levels and aggregation percentage (*P* < 0.05; *n* = 8). These results suggest that patients accumulate more levels of everolimus within the first months after transplantation, and elevated circulating everolimus have a negative effect in platelet function.

## Discussion

Rapamycin-based therapies represent a good alternative in cardiac-transplanted patients, in patients presenting cardiovascular complications and particularly, in those patients where immunosupression-related neoplasias have been diagnostized. Despite the information concerning the effects of mTOR inhibitors in platelets function is scarce, the reports available in the literature regarding sirolimus effects in platelets function are controversial.

Our results indicate that long-term mTOR inhibitors administration alters Ca^2+^ release and impairs Ca^2+^ entry in response to Thr and also dependent of acidic store depletion using TBHQ. Contrary, in kidney-transplanted patients under sirolimus medication no significant alteration has been observed in Ca^2+^ homeostasis induced by ADP in platelets. Furthermore, everolimus modified the Ca^2+^ homeostasis pattern induced by all stimuli used.

Interestingly, the alteration observed in Ca^2+^ homeostasis resulted most evident in patients treated with sirolimus during 24–26 months; after that, most patients included in this study recovered Ca^2+^ mobilization patterns in response to Thr. This finding explains the high correlation coefficient found between Ca^2+^ entry levels and time medicated. In this sense, rapamycin-dependent inhibition of SOCE evoked by the SERCA inhibitor, cyclopiazonic acid, has been recently reported in pulmonary vascular cells 43, and in human pulmonary arterial smooth muscle cells, suggesting that mTOR is downstream to PDGF receptor participating in the association between STIM1/Orai and subsequently regulating SOCE 44.

Recent studies have shown that platelet aggregation is enhanced in response to ADP 45. Furthermore, other studies have proposed that sirolimus might enhance cyclooxygenase activity, as they presented evidence for a reduced aspirin effect by increasing rapamycin concentration in ‘*in vitro*’ assay using shorter incubation time, which is different to a cronical exposition during years presented here 45. More specific mTOR inhibitors, like PP242 and torin1, enhanced platelet aggregation in response to SFLLRN, a PAR-1 receptor agonist. Moreover, platelets incubated for 15 min. with rapamycin (200 nM) does not reported significant alteration in aggregation activated by SFLLRN, suggesting that under these experimental conditions mTOR2 regulates Akt function and subsequently platelet activity, through this pathway that would be non-sensitive to rapamycin 1, 14. In our hands, Akt resulted dephosphorylated during the initial steps of platelets activation, which would allow cytoskeleton reorganization. After the initial steps of platelet activation, Akt would probably become highly phosphorylated as reported in a very recent publication, in which the author evaluated phospho-Akt upon 15 min. of stimulation with Thr 42. We have not found changes in Akt phosphorylation pattern between healthy individual and patients belonging to group II, which would indicate that mTOR1 would not be involved in the modification of Akt activity, as previously reported 42.

However, a recent study has reported that mTOR2 is sensitive to rapamycin depending on the time of exposure to the drug 46. In this sense, our findings are unlikely explained by the exclusive participation of mTOR2 but also to the participation of mTOR1 instead, since mTOR1 downstream protein kinase, p70S6K1 has been presented previously regulating Bcl-3 secretion of glycoprotein αIIb/β3 evoked by Thr, which is required for platelets response to fibrinogen, von Willebrand factor, vitronectin and fibronectin 47–49. We suggest that this controversy relies in the agonist used for stimulating platelets, since despite stimulating platelets with Thr, after granules secretion other autocrine stimuli, like ADP or serotonin, might modify the initial stimulation evoked by Thr, or by contacting with disrupted vessel exposing collagen, vWF, etc.

Time-dependent membrane exposure of P-selectin, as well as a reduced δ-granule secretion containing autocrine and paracrine substances, is presented here. Using cytometry we have evaluated resting platelets (CD41+ cells) and Thr-stimulated platelets from sirolimus-treated kidney transplant patients. In our patients, an elevated degranulation is observed in resting platelets, which might explain the reduced response and impaired aggregation found upon application of external stimulus presented above.

Finally, as first clinical trials were set up, thrombocytopenia is the most evident side-effect reports in patients under rapamycin medication, or rapamycin analogues administration 50, 51. We have observed that almost half of our patients presented very low platelet count, and even some of them presented severe thrombocytopenia. Enhanced clearance of pre-activated platelets or reduced platelets generation from bone marrow alteration, leads to thrombocytopenia. These hypotheses are under an intense investigation nowadays, revealing that mTOR might play an important role in platelet production 52–54.
